# Precision Treatment of Anthracycline-Induced Cardiotoxicity: An Updated Review

**DOI:** 10.1007/s11864-024-01238-9

**Published:** 2024-07-27

**Authors:** Ziyu Kuang, Yuansha Ge, Luchang Cao, Xinmiao Wang, Kexin Liu, Jiaxi Wang, Xiaojuan Zhu, Min Wu, Jie Li

**Affiliations:** 1 Department of Oncology, Guang’anmen Hospital, China Academy of Chinese Medical Sciences, Beijing, 10053 China; 2 Graduate School, Beijing University of Chinese Medicine, Beijing, 10029 China; 3 Department of Cardiovascular, Guang’anmen Hospital, China Academy of Chinese Medical Sciences, Beijing, 10053 China; 4 The 3rd affiliated hospital of Zhejiang Chinese Medical University, Hangzhou, 310005 China

**Keywords:** Anthracycline, Cardiotoxicity, Cancer, Chemotherapy, Doxorubicin, Heart failure

## Abstract

Anthracycline (ANT)-induced cardiotoxicity (AIC) is a particularly prominent form of cancer therapy-related cardiovascular toxicity leading to the limitations of ANTs in clinical practice. Even though AIC has drawn particular attention, the best way to treat it is remaining unclear. Updates to AIC therapy have been made possible by recent developments in research on the underlying processes of AIC. We review the current molecular pathways leading to AIC: 1) oxidative stress (OS) including enzymatic-induced and other mechanisms; 2) topoisomerase; 3) inflammatory response; 4) cardiac progenitor cell damage; 5) epigenetic changes; 6) renin-angiotensin-aldosterone system (RAAS) dysregulation. And we systematically discuss current prevention and treatment strategies and novel pathogenesis-based therapies for AIC: 1) dose reduction and change; 2) altering drug delivery methods; 3) antioxidants, dexrezosen, statina, RAAS inhibitors, and hypoglycemic drugs; 4) miRNA, natural phytochemicals, mesenchymal stem cells, and cardiac progenitor cells. We also offer a fresh perspective on the management of AIC by outlining the current dilemmas and challenges associated with its prevention and treatment.

## Introduction

Since the 1990s, the mortality rate of cancer has gradually decreased, while the incidence rate has steadily increased. Consequently, the side effects related to malignant tumor treatment have increasingly gained attention. According to epidemiological data, two of the main causes of illness and death globally are cardiovascular diseases and cancer [1]. Anti-cancer therapies, including chemotherapy, targeting, immunotherapy, and radiotherapy, can elevate the risk of cardiovascular diseases during and post-treatment, leading to cancer therapy-related cardiovascular toxicity (CTR-CVT) [2].

Anthracycline (ANT)-induced cardiotoxicity (AIC) is a particularly prominent form of CTR-CVT. Many cancers, including leukemia, lymphoma, sarcoma, and breast cancer, are commonly treated with ANTs, such as doxorubicin (DOX), epirubicin, daunorubicin, and norerythromycin [3]. However, the safety of ANTs has been a topic of ongoing concern, with the most significant clinical adverse events being the increased incidence of cardiovascular toxicity [3]. The occurrence of congestive heart failure associated with ANTs is 2–4%, sub-clinical structural changes occur in approximately 10%, arrhythmias occur in 12%, and cardiac-related biomarker positivity is observed in 30–35% of patients [4]. Moreover, the cardiac dysfunction caused by ANTs is often progressive and irreversible, with noticeable ultrastructural abnormalities in the myocardium. As new anti-tumor drugs emerge and AIC garners more attention, particularly in patients at high cardiovascular risk, the National Comprehensive Cancer Network (NCCN) guidelines have begun to restrict the use of ANTs chemotherapy regimens [5]. Nonetheless, ANTs remain a vital component in the clinical treatment of cancer. Therefore, it is of great importance to delve into the molecular mechanisms and clinical prevention and treatment of AIC.

To date, the precise mechanisms underlying AIC are not fully understood. However, it is crucial for the treatment of AIC. To determine effective clinical prevention and treatment methods, we have reviewed the latest advancements in the pathogenesis and therapeutic strategies for AIC.

## Overview of Mechanisms of AIC

**Fig. 1:**
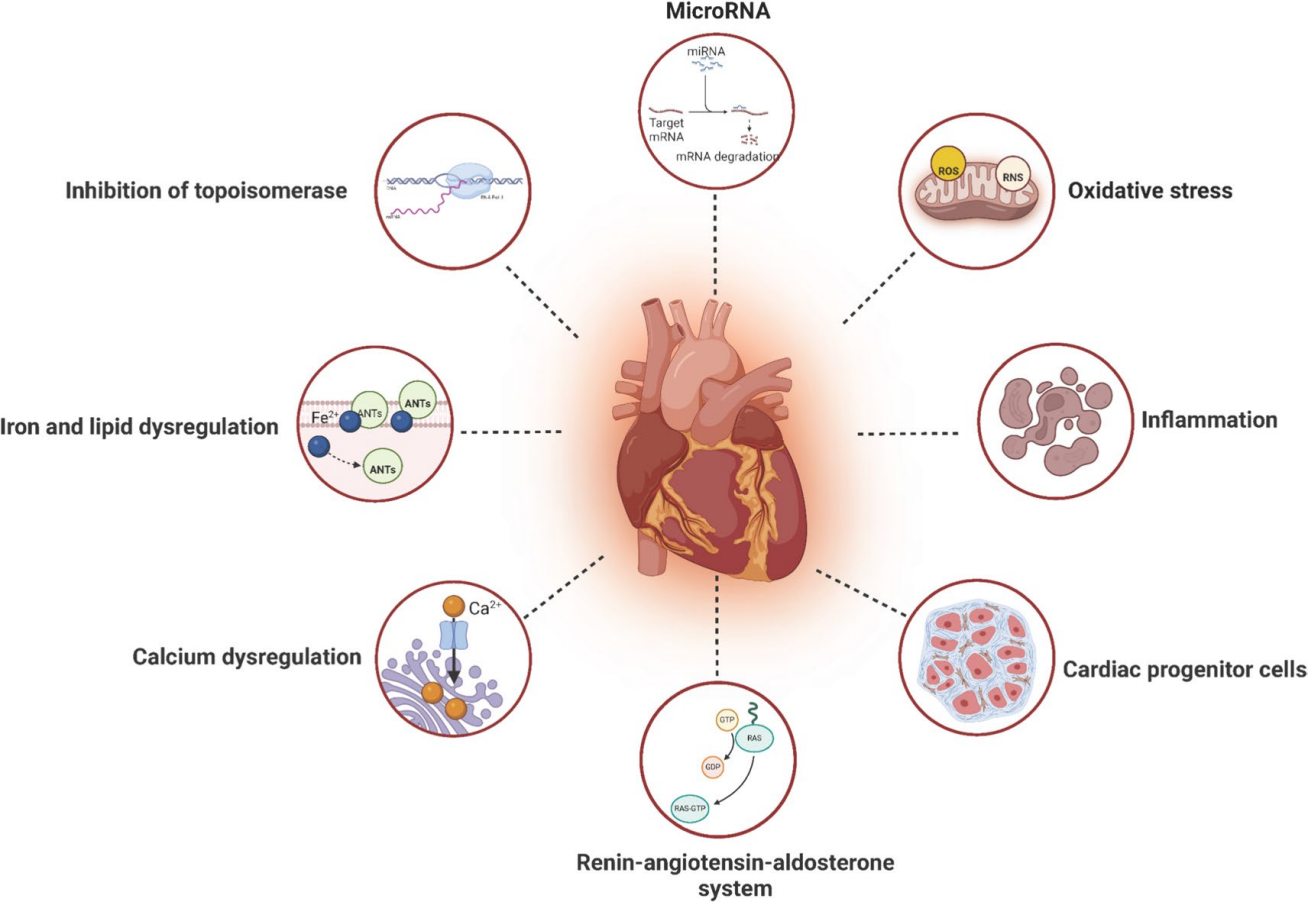
Mechanisms of AIC with pharmacologic targets (Created with BioRender.com)

The potential mechanisms of AIC have not been completely elucidated, oxidative stress and the action of topoisomerases are two well-established mechanisms.. In addition, other mechanisms of AIC are continually being explored. The cardiac toxicity caused by ANTs may be due to complex and multi-factors, with cross-talk and synergistic effects among various mechanisms contributing to AIC (Fig. 1).

### Oxidative Stress

**Fig. 2:**
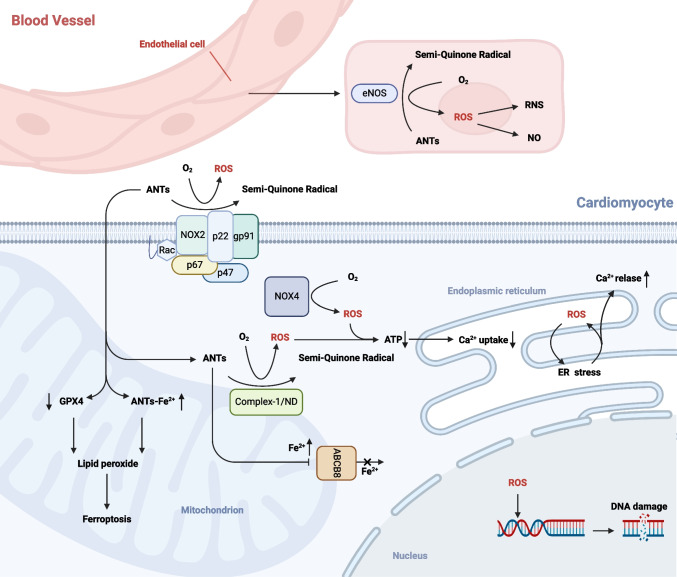
Mechanisms of oxidative stress in AIC (Created with BioRender.com)

The most well-researched mechanism of AIC, Oxidative stress (OS), is caused by an imbalance between the body's deteriorating antioxidant defense system and the increased production of reactive oxygen species (ROS) and reactive nitrogen species (RNS). One-electron reduction products of oxygen, or ROS, are produced when electrons escape the respiratory chain and take up to 2% of the oxygen in the body before failing to reach the terminal oxidase. These include the one-electron reduction product superoxide anion (O^2^·-), the two-electron reduction product hydrogen peroxide (H_2_O_2_), and the three-electron reduction product nitric oxide (NO) and hydroxyl radical (·OH). Although enzymes play a major role in the creation of ROS, additional processes can also contribute to the development of ROS and OS. Overexposure to ROS oxidizes lipids, proteins, and nucleic acids, leading to permanent damage to biological components including DNA and cell membranes [6] (Fig. 2).

#### Enzymatic Oxidative Stress

ANTs promote the formation of ROS through a variety of enzyme pathways,including actions on nitric oxide synthase (NOS), nicotinamide adenine dinucleotide phosphate (NADPH) oxidase (NOX), and the reduced form of nicotinamide-adenine dinucleotid (NADH) dehydrogenase (ND) of mitochondrial electron transport chain complex-I.

The majority of energy in all tissues of the human body comes from oxidative phosphorylation of aerobic metabolism inside the mitochondria, especially the cardiac tissue [7]. Complex-I/ ND is the first enzyme in the respiratory chain. The ANTs redox cycle mainly occurs on Complex-I in the mitochondrial respiratory chain [8, 9]. ND partly converts the quinone moiety of ANTs to semi-quinone radical production in cardiomyocytes. Semi-quinones auto-oxidize to create parent ANTs and superoxide anions when molecular oxygen is present [4]. The buildup of superoxide anions and decreased adenosinetriphosphate (ATP) synthesis are the outcomes of this self-sustaining redox cycle of ANTs in ND. Superoxide dismutase (SOD) has the ability to spontaneously convert superoxides to H_2_O_2_. This mechanism explains how ANT-induced mitochondrial-associated ROS production can result in heart failure and cellular death [7].

The NOX, is a complex multicomponent protein, and it is closely related to the production of ROS in the cardiovascular [10]. There are seven subtypes of NOX [11]. The two main NOX subtypes that are expressed in cardiomyocytes are organel-bound NOX4 and cell-membrane-bound NOX2, which is a mechano-sensor in cardiomyocyte furthermore [12]. NOX2 is made up of the guanosine triphosphate binding protein (Rac1), the cytoplasmic regulatory subunits p47phox, p67phox and p40phox, a heterodimer of gp91phox and p22phox, and membrane-bound cytochrome b-558 [13]. These enzymes facilitate the semi-quinone radical's formation, which is essential to AIC, by transferring an electron from NADPH to ANTs, and the leading to the production of ROS [14]. Studies have shown that NOX2-lacking or gp91phox knockout mice are resistant to DOX-induced cardiotoxicity (DIC), exhibiting a decrease generation of superoxide radicals in heart tissue. In contrast, wild-type mice exhibited cardiac dysfunctions, including myocardial atrophy, programmed cell death of cardiomyocytes, and interstitial fibrosis [10, 15, 16].

Three subtypes of the NOS enzyme class—neuronal NOS (nNOS), endothelial NOS (eNOS), and injury-inducible NOS (iNOS)—catalyze the production of NO from L-arginine in vivo [17]. The majority of eNOS is present in vascular endothelial cells. OS can be caused by ANTs binding to the reductase region of eNOS and reducing the quinone moiety to semi-quinone radicals. This produces superoxide radicals and hydrogen peroxide. Nitrosation stress is the outcome of this as well as decreased NO synthesis and the production of peroxynitrite (reactive nitrogen species, or RNS) [18–23]. RNS have been shown to be a trigger for DIC death [18]. In addition to its association with cardiomyocytes, eNOS plays a particularly significant role in the pathophysiology of ANT-associated endothelial toxicity.

#### Other Mechanisms of Oxidative Stress

A important contributing factor to AIC is iron imbalance. ANTs cause ROS generation go through a number of redox processes, and generate semi-quinone metabolites or Doxorubicinol Hydrochloride, increasing the level of DOX-Fe^2+^ complexes and then OH- [24]. Lipid peroxidation caused by DOX can result in cardiotoxicity [25], wihle ferroptosis is the outcome of lipid oxidation. The ROS generated from ANTs metabolism can form several products, such as 4-hydroxynonenal (4-HNE), through the lipid peroxidation of unsaturated fatty acids [26]. 4-HNE is a critical marker of ferroptosis. Glutathione peroxidase 4 (GPX4) serves as an endogenous lipid peroxidase scavenger and is a pivotal regulator of ferroptosis. Research indicates that ANTs down-regulates GPX4, leading to untimely clearance of lipid oxidation and further inducing mitochondria-dependent ferroptosis [24]. On the other hand, ANTs contribute to the accumulation of iron in cardiomyocytes. DOX regulates the ATP-binding box ABCB8 protein (a mitochondrial iron-exporting protein) [27], resulting in decreased protein levels and consequently impeding the iron export process in mitochondria [28].

ANTs can cause disruptions in calcium homeostasis and cardiac systolic dysfunction [29]. ANTs cause endoplasmic reticulum (ER) stress, decrease mitochondrial respiration, produce ROS from mitochondria and NOX, change Ca^2+^ signaling, and cause DNA damage in cardiomyocytes [30]. Mitochondrial dysfunction, decreased ATP synthesis, and decreased Ca^2+^ uptake are the results of ANTs' effects on the electron transport chain and increased creation of ROS. An increase in ROS causes ER stress, and when cytoplasmic Ca^2+^ concentration rises due to ER Ca^2+^ leakage, intracellular Ca^2+^ homeostasis is upset, cardiac triggering activity increases, and atrial fibrillation occurs Furthermore, phosphorylation of calcium regulatory protein, which causes sarcoplasmic reticulum calcium leakage, and activation of calcium/calmodulin-dependent protein kinase II increase late sodium current and L-type calcium current in atrial myocytes are significant factors in the development of atrial fibrillation [31].

### Inhibition of Topoisomerase

As a topoisomerase inhibitor, ANTs (e.g. DOX) could inhibit Top 2 by forming a covalent Top2-DOX complex, resulting in double-stranded DNA breaks [32, 33]. Top 2 consists of isoenzymes Top 2α and Top 2β. Top 2α is highly expressed in proliferating (malignant and non-malignant) cells during G2/M phase [34–36] and is essential for chromosome separation [37]. ANTs chemotherapy has shown high efficacy because Top 2α is up-regulated in cancer cells, in contrast, Top 2β is the sole Top 2 present in cardiac tissue [38]. ANTs are inserted into cardiomyocyte DNA by Top 2β, leading to DNA damage in cardiac tissue. Moreover, ANTs downregulates the expression of multiple genes through inhibiting Top 2β, including those involved in mitochondrial biogenesis and antioxidant function, which can protect cardiomyocyte from damage [39–41]. So the inhibition of Top2β can promote the AIC.

### Inflammatory Response

ANTs causes oxidation and ER stress, which results in cell necrosis and apoptosis. It also encourages the release of damage-associated molecular patterns, which increases the expression of markers associated with pyroptosis (cysteiny l aspartate specific proteinase 1 [caspase-1], Interleukin-1β [IL-1β], and IL-18), inflammatory body markers (Toll-like receptor 4 [TLR4] and NOD-like receptor thermal protein domain associated protein 3), cell signaling proteins, tumor necrosis factor (TNF), and proinflammatory M1 macrophages [42–44]. These lead to pyroptosis of cardiomyocytes and the formation of AIC.

### Cardiac Progenitor Cell Damage

Cardiac progenitor cells (CPC) can differentiate into smooth muscle cells, endothelium cells, and cardiomyocytes, and they also exhibit the stem cell antigen c-kit (proto-oncogene protien) and are capable of self-renewing, clonality, and pluripotency [45]. ANTs promote oxidative stress, and DOX causes an average 30% shortening of CPC telomeres and inhibition of cell cycle through p53 activation, collectively inhibiting CPC growth and survival [46]. Furthermore, DOX has been found to induce the rapid translocation of nucleolar protein and nuclear phosphoprotein to the nucleoplasm and inhibit the synthesis of new pre-ribosomal RNA, resulting in CPC damage through nucleolar stress [47]. CPC damage may be a essential cause of impaired cardiomyocyte renewal, accumulation of senescent cells, apoptosis, and the onset of ventricular dysfunction, supporting the notion that progenitor cell dysfunction may influence the development of cardiomyopathy in vivo.

### Epigenetic Changes

Epigenetics refers to modifications in gene expression or cellular phenotype that occur without altering the DNA sequence. These modifications include histone modification, DNA methylation, and non-coding RNA (ncRNA). Histone modifications, ncRNA expression, and DNA methylation have all been linked to AIC [48, 49]. Research has found that rats lacking methyl donors developed cardiomyopathy, with a breakdown in mitochondria arrangement in heart muscle [50]. In rat hearts treated with DOX, global hypomethylation of DNA has been detected, along with dysregulated expression of the mitochondrial gene products encoded by the nuclear and mitochondrial genomes [51]. Histone modification is also involved in AIC [52–54]. Furthermore, the control of ncRNAs, such as microRNA (miRNA), circle RNA (circRNA) and long non-coding RNA (lncRNA), is another known epigenetic modification. miRNA regulates the cardiovascular system and may contribute to DIC by causing damage to the cardiomyocytes via a number of different mechanisms. Recent research has demonstrated that deregulation of miRNA expression might worsen the pathological processes associated with DIC, such as OS induction, apoptosis, ion channel failure, and microvascular dysfunction. A meta-analysis found that the following genes have been associated with AIC during chemotherapy in breast cancer patients: miR-1, miR-133, miR-126, and let-7f, etc. [55].

### RAAS System Dysregulation

Numerous forms of cardiovascular remodeling are associated with activation of the renin–angiotensin–aldosterone system (RAAS). Elevated levels of angiotensin II (Ang II) have the ability to cause OS in cardiomyocytes, which in turn promotes necrosis and apoptosis via the mitochondrial route. Research has demonstrated that AngII-1A receptor (AT1) knockout mice had much better cardiac function than WT mice. Histological analysis has revealed that DOX causes the loss of myofibrillar fibers in WT mice and increase the number of apoptotic cells, with the AT1-mediated Ang II signaling pathway playing a significant role in DOX-induced heart damage [56]. Moreover, Ang II can worsen cardiac function and exacerbate cardiac fibrosis in DOX-exposed mice, with a significant increase in multiple inflammatory and fibrosis markers [57].

## Current Prevention and Treatment Strategies of AIC

Some of the strategies that have been studied include reducing the probability of AIC by adjusting the infusion timing, delivery mode (e.g., liposomal DOX), and dosage of ANTs. Dexrazoxane (DEX) is a cardioprotective agent approved for patients at high risk for AIC or those who have received substantial cumulative doses of ANTs [58]. Additionally, drugs targeting RAAS system and glucose-lowering drugs are utilized for secondary prevention of AIC [59].

### Dose Reduction and Change of Single Infusion Dose

Dosage is a critical factor influencing AIC. A previous study observed a clear correlation between the occurrence of DIC and the total dose administered, and it is therefore recommended that the dose of DOX-based regimens should not exceed 550 mg/m^2^ and that of epirubicin should not exceed 900 mg/m^2^ [60]. The 2022 European Society of Cardiology (ESC) Guidelines consider DOX or equivalent doses ≥ 250 mg/m^2^ as high risk (Table 1 shows the high-risk doses of other ANTs based on equivalent doses of DOX in the ESC guidelines) [61]. Because of the linear relationship between ANT infusion rate and AIC, slower continuous infusion doses may reduce the risk of AIC compared with rapid administration [62], and a meta-analysis showed that ANTs with infusion intervals of six hours or more were associated with a reduced risk of heart failure (RR: 0.27, 95% CI: 0.09–0.81) and subclinical AIC (RR: 0.35, 95% CI: 0.15–0.9) compared with high-dose administration [63].

**Table 1:** High-risk doses of other ANTs at the same dose of DOX conversion

	Doxorubicin	Epirubicin	Daunorubicin	Mitoxantrone	Idarubicin
AIC dose ratio	1.0	0.8	0.6	10.5	5
Isoequivalent dose	100 mg/m^2^	125 mg/m^2^	167 mg/m^2^	9.5 mg/m^2^	20 mg/m^2^

### Altering Drug Delivery Methods

Due to the irreversibility of AIC, researchers have made some efforts to address these problems and have tried to establish a number of novel drug delivery systems (DDSs) to develop delivery methods, among which liposomes are an advantageous DDS. Compared with direct administration, liposomal delivery systems have significantly improved the efficacy and safety of chemotherapeutic drugs, such as pegylated liposomal DOX, DOX hydrochloride liposomes, etc. [64]. A meta-analysis showed a significant increase in cardiotoxicity in conventional DOX compared to liposome-encapsulated DOX (RR: 3.75, 95% CI: 2.46–5.70) [65]. Polymeric nanoparticles (NPs) are also being explored for drug delivery to malignant tumors, where DOX, when loaded onto NPs, is targeted to tumour tissue and prevented from accumulating in non-target organs to reduce its adverse effects. For example, Live Macrophage-Delivered DOX-Loaded Liposomes further increases the cumulative concentration of ANTs at the tumor site compared to conventional liposome delivery, allowing the drug to penetrate deeper into the tumor tissue, and in addition, the delivery system uses highly biocompatible materials with a lower incidence of AIC without compromising anti-tumor efficacy [66]. Santin et al. [67] designed poly(lactic-co-glycolic acid)-grafted silica NPs (PLGA-NPs). In terms of mechanism, PLGA-NPs reduce DOX-induced lysosomal alkalinization in cardiomyocytes in the presence of DOX, thereby improving cardiomyocyte lysosomal function and autophagic flux, and alleviating DOX-related mitochondrial dysfunction and OS.

### Antioxidants

A number of studies have indicated that multivitamins (e.g., B vitamins, vitamin C, vitamin D, etc.) may act as antioxidants in the mitigation of AIC. Vitamin B6 can counteract OS caused by ANTs by reducing the expression of the Na + /H + exchanger, lowering serum malondialdehyde (MDA) and elevating serum SOD levels, and lowering the ratio of B-cell lymphoma-2 (Bcl-2)-associated X protein (Bax) [68]. Nicotinamide adenine dinucleotide, nicotinamide adenine dinucleotide phosphate, and its reduced form NAD(P)H are two common functional cofactors for vitamin B3. Known as the NAD(P)(H) pool, these cofactors are closely linked to every vital bioenergetic, anabolic, and catabolic pathway [69]. Furthermore, via controlling many intracellular Ca^2+^ signaling pathways, mitochondrial respiration, and ATP synthesis, the NAD(P)(H) pool also plays a critical role in cellular metabolism and cell signaling [70]. It has been established that vitamin C can guard against AIC, and that sodium-dependent vitamin C transporter-2 is less expressed and localized in cardiac tissue when exposed to DOX. But it has been shown that taking supplements of vitamin C can undo this alteration [71]. Simultaneously, vitamin C can inhibit the levels of cardiac pro-inflammatory cytokines IL-1β, TNF-α, and IL-6, as well as the inflammatory response in cardiac tissue. It can also downregulate the increase in the expression of pro-apoptotic proteins Bax, Bcl-2/adenovirus E1B19kDa interacting protein 3, Bcl-2 antagonist killer, and caspase-3, NO and NOS activities, protein nitrosylation, and inducible NOS protein expression caused by DOX [72]. 4-HNE, NAD(P)H dehydrogenase quinone 1 (NQO1), and other lipid peroxidation markers of ferroptosis are of significant importance. The simultaneous use of vitamin D and DOX (10 mg/kg) has been observed to result in a reduction in the phosphorylation levels of 4-HNE and NQO1 in heart tissues when compared to DOX alone.This has been demonstrated to inhibit OS and ferroptosis [73].

Glutathione (GSH) is the most crucial antioxidant in redox homeostasis. It does this by using both enzymatic and non-enzymatic antioxidants, such as glutathione transferases, catalase (CAT), SOD, and GPX, which are in charge of detoxifying endogenous substances like lipid hydroperoxide, superoxide, etc. [74]. The administration of DOX in mice has been shown to significantly enhance left ventricular ejection fraction (LVEF) and reduce the level of brain natriuretic peptide (BNP) when combined with GSH [75].

Coenzyme Q10 is a potent antioxidant and free radical scavenger. Following coenzyme Q10 treatment, the activity of SOD was significantly increased. Additionaly, hematoxylin–eosin staining demonstrated a notable reduction in the number of autophagosomes in rats following coenzyme Q10 treatment, when compared with rats treated with DOX alone [76]. In a separate study, the DOX plus Q10 group exhibited increased CAT activity (*P* < 0.05) and decreased MDA concentrations (*P* < 0.05) in comparison to the DOX group, thereby antagonizing OS levels [77].

### Dexrezosen and its Analogues

The only cardioprotective medication licensed by Food and Drug Administration to decrease AIC is the iron chelator bisdioxopiperazine agent DEX [78]. DEX has been officially approved for adult patients with advanced or metastatic breast cancer who have received DOX or equivalent drugs with a minimum cumulative anthracycline dose of 300 mg/m^2^ [79] and as a primary prevention strategy for AIC. The antagonism of AIC by DEX is generally based on 3 mechanisms: First, DEX protects cardiomyocytes by hydrolyzing into the iron-chelating metabolite ADR-925 (N, N'-[(1S)-1-methyl-1,2-ethanediyl]-bis [(N-(2-amino-2-oxoethyl)]glycine) [80]. This metabolite can then be intracellularly converted into a ring-opening chelator. This is the mechanism of action of DEX. For instance, in DIC, ADR-925 displaces iron within the DOX-Fe^3+^ complex and binds to iron, which can impede iron-mediated free radical production, obstruct the inactivation of respiratory enzymes by iron complexes, and reduce DIC. Secondly, DEX is a topo IIβ inhibitor, and DEX treatment results in a near complete loss of topo IIβ in cardiomyocytes, a process that approximates exponential decay (t_1/2_ = 2.7 h) [81]. The action of this mechanism results in the reduction of ANT-induced formation of topo IIβ-hidden DNA double-strand breaks, thereby reducing cardiomyocyte damage. DEX analogues also have antagonistic AIC effects, such as meso-derivative 11 (ICRF-193), a DEX analogue, which inhibits and depletes topo IIβ in cardiomyocytes more effectively than DEX and shows the highest cardioprotective efficiency. Notably, the cardioprotective effect of ICRF-193 does not interfere with the antitumor activity of ANTs [82]. Nevertheless, there is still a lack of consensus regarding the inhibition of ADR-925 hydrolysate on topo IIβ. For example, Jirkovský et al. [83] demonstrated that DEX inhibits and depletes topo IIβ, thereby preventing daunorubicin-induced heart damage. In contrast, ADR-925 does not alleviate daunorubicin-induced heart damage. Thirdly, DEX has been demonstrated to antagonize programmed cell death of cardiomyocytes (e.g., ferroptosis). Research has revealed that when cardiomyocytes are treated with DOX, there is a considerable rise in p38(a kind of MAPK) mitogen-activated protein kinase (MAPK) phosphorylation [84]. DEX has been shown to inhibit DOX-induced phosphorylation of p38MAPK and p65, and to down-regulate the expression of the p38MAPK/nuclear factor-k-gene binding (NF-κB) pathway in mouse hearts [85], inhibiting DOX-induced cardiomyocyte necrosis and apoptosis.

### Statins

As hydroxymethylglutaryl coenzyme A (HMG-CoA) inhibitors, statins have long been considered one of the most effective treatments for lowering cardiovascular events [86]. Clinical studies and mechanistic studies on statin prophylaxis and treatment of AIC are ongoing. In a clinical trial with 300 lymphoma patients receiving ANT therapy, patients on atorvastatin experienced a decreased frequency of AIC (9% versus 22%, P = 0.002) and a higher rate of loss in LVEF in the placebo group (RR: 2.9; 95% CI: 1.4–6.4) [87]. In terms of mechanism, survivin is a member of the apoptosis inhibitory protein family and achieves cardioprotective effects through the Forkhead box O1 /signal transducer and activator of transcription 3 (STAT3) /surfactant protein 1 transcriptional network. Statins reduce DIC by transcriptionally regulating the anti-apoptotic protein survivin [88]. According to a different research, atorvastatin also reduced myocardial fibrosis and myocardial apoptosis via modifying phosphorylated protein kinase B (p-Akt), heat shock 70 kDa protein, phosphorylated c-Jun amino-terminal kinase (p-JNK), and phosphorylated extracellular signal-regulated kinase (p-ERK) signaling [89]. Rosuvastatin, as another commonly used inhibitor of HMG-CoA, downregulates the levels of cTnI and LDH and inhibits OS and inflammatory processes such as MDA and IL-17 [90].

### RAAS Inhibitors

The 2022 ESC guidelines additionally advocate the use of beta-blockers (BBs), angiotensin receptor blockers (ARBs), and angiotensin-converting enzyme inhibitors (ACEIs) for the secondary prevention of ANT cardiotoxicity in patients with AIC [61]. These drugs have been demonstrated to facilitate ventricular recovery by inhibiting ventricular remodelling mediated by adrenergic and neuroendocrine disorders. Furthermore, several meta-analyses have indicated that prophylactic use reduces the incidence of AIC [91, 92]. In terms of mechanism, Lódi et al. [93] showed that in animal models treated with DOX, both ACE inhibitors (bisoprolol) and BB (perindopril) inhibited cardiomyocyte apoptosis, while preventing DOX-induced fibrotic remodeling and DOX-induced increase in caspase-3 levels, which allowed for the preservation of myocardial ultrastructure [93]. Benazepril hydrochloride pretreatment counteracts DOX-induced OS and inhibits the activation of apoptosis via the phosphatidylinositol 3-kinase(PI3K)/Akt signalling pathway [94]. DOX increased the production of ROS in H9c2 cells and up-regulated the expression of AngII type I receptor, NOX2, NOX4, caspase-3, caspase-9, and MAPK signalling proteins, including p-p38, p-JNK, and p-ERK. The administration of valsartan was found to attenuate these effects [95]. Besides, valsartan markedly reduced the expression levels of several proteins linked to ER stress and apoptosis, such as caspase-3, activating transcription factor (ATF)-6, ATF-4, eukaryotic initiation factor (eIF)-2α, Bax, C/EBP homologous protein (CHOP), pancreatic endoplasmic reticulum kinase (PERK), iron responsive element-1α, and glucose-regulated protein 78 (GRP78) [96]. However, the clinical evidence supporting the use of ACEIs/ ARBs/ BBs to prevent the cardiovascular toxicity of ANTs remains inconclusive. In particular, the basic assessment of whether the application of cardioprotective therapy in low-risk patients is beneficial remains a matter of contention [97–100].

### Hypoglycemic Drugs

The use of hypoglycemic drugs can mitigate the risk of AIC in diabetic patients to a certain extent. Metformin (MET) could exerts its cardioprotective effects by acting on the adenosine monophosphat-activated protein kinase (AMPK) pathway, which regulates the occurrence of mitochondrial biological processes through peroxisome proliferator-activated receptor gamma coactivator 1α signaling, reduces apoptosis by inhibiting the mammalian target of rapamycin (mTOR) signalling, increases autophagy through Unc-51-like kinase 1, and reduces fibrosis by inhibiting transforming growth factor (TGF)-β signalling. In vivo and in vitro studies have demonstrated that MET prevents DOX-induced cleavage of caspase-3 and Bax increases. MET also prevents the downregulation of Bcl-2, activates the AMPK pathway, while inhibiting ferroptosis and improving cardiac function by activating AMPKα2 phosphorylation [101, 102]. However, the results of clinical studies appear to be conflicting, with a 143-person RCT showing that metformin did not prevent the development of AIC compared with placebo [103]. Nevertheless, a different clinical trial revealed that after a year of starting ANT medication, patients treated with metformin had a reduced incidence of heart failure (3.6% vs. 10.5%; P = 0.022), and metformin (HR: 0.71; 95% CI: 0.50–1.00; P = 0.049) was also linked to a lower death rate [104]. Consequently, further validation is required with regard to the benefits of MET in patients with ANTs.

On the other hand, research on the prevention and treatment of AIC using sodium-glucose cotransporter 2 (SGLT-2) inhibitors has shown that dapagliflozin (DAPA) can counteract AIC by reducing OS through PI3K/Akt/nuclear respiratory factor (Nrf) 2 signalling and by preventing the downregulation of markers linked to fibrosis (phosphorylated collagen I, α-smooth muscle actin, fibronectin, and Small mothers against decapentaplegic [SMAD] 3) and hypertrophy (atrial natriuretic peptide and BNP) [105]. Furthermore, another in vivo study showed that DAPA decreased cardiac expression of Bax and caspase-3,while increasing Bcl-2 expression. Additionally, DAPA also significantly reduced ER stress-related proteins, including ATF-4, PERK, CHOP, eIF-2α, and GRP78 [106]. Nevertheless, there is a paucity of clinical studies on SGLT-2 inhibitors.

## Novel Therapies for Pathogenesis-Based AIC

As ncRNAs have emerged as a research focus in transcriptomics in recent years, and cell therapies and natural pharmaceutical ingredients have also been demonstrated to be effective in intervening in AIC, it may be concluded that these approaches could be effective in preventing and treating AIC.

### miRNAs

The most extensively researched role in AIC is that of miRNAs, and the expression of certain miRNAs is dependent on the regulatory function of other ncRNAs, including circRNAs and lncRNAs. It has been demonstrated that miRNAs are essential to the cardiovascular system and can either directly or indirectly control the onset and progression of AIC. miR-1, miR-133 family are the most explored ncRNAs in cardiovascular system, [107, 108], In addition, some miRNAs, such as miR-15, miR-22, miR-30, miR-34 family are important for the physiology and pathology of the cardiovascular system. In addition to acting as regulators of AIC, miRNAs also serve as regulatory targets for other ncRNAs, such as lncRNAs and circRNAs, which promote or inhibit the occurrence of AIC by acting as sponges for multiple miRNAs. The specific roles played by miRNAs in AIC are presented in Table 2.

**Table 2:** Therapeutic role of miRNAs in AIC

ncRNAs	Promote/inhibit AIC	Target	Effect	References
miRNA regulates AIC directly				
miR-15b-5p	promote	Bcl-2, Bax, Akt, Bmpr1a	exacerbate DOX-induced apoptosis	[109]
miR-22	promote	SIRT 1,	exacerbate cardiomyocyte apoptosis	[110]
miR-30	inhibit	GATA-6	lead to dilated, hypertrophic cardiomyopathy	[111]
miR-34-5p	promote	SIRT3	lead to pyroptosis by regulating autophagy and mitochondrial ROS	[112, 113]
miR-130a	promote	PPARγ	exacerbate cardiomyocyte apoptosis, inflammation	[114]
miR-140-5p	promote	Nrf2, Sirt2	OS	[115]
miR-146a	inhibit	P53, TAF9b	inhibits apoptosis and partially reverses DIC	[116]
regulate AIC indirectly through miRNAs				
lncRNA NEAT1	inhibit	miRNA let-7f-2-3p	-	[117]
lncRNA MALAT1	inhibit	miR-92a-3p, ATG4a	improve mitochondrial metabolism	[118]
circITCH	inhibit	miR-330-5p	regulate Ca^2+^	[119]
circArhgap12	promote	miR-135a-5p	stimulate OS and apoptosis	[120]

### Natural Phytochemicals

Natural substances found in herbs and plants called phytochemicals have been identified as possible treatments for AIC [121]. Research has demonstrated that when paired with DOX, the multi-activity and multi-targeting of phytochemicals including ginsenoside Rg1, paeonol, and tanshinone I can provide greater clinical benefits. The specific roles played by different types of natural phytochemicals in AIC are presented in Table 3.

**Table 3:** Therapeutic role of natural phytochemicals in AIC

Type	Origin	Target	Effect	Advantage and disadvantage	References
Paeonol	Paeonia officinalis	STAT3, Mfn2, PKCε, Notch1, miR-1, PI3K, AKT, mTOR, NF-κB	prevent hypertrophic responses, improve heart failure, mitigate histopathological alterations, decreas inflammation, myocardial apoptosis, and enhance autophagy	not interfere with the anti-tumor efficacy of DOX; short half-life, limited bioavailability, and poor stability	[122–125]
Scutellarin	Scutellaria baicalensis	TGF-β1, Smad2, AMPK, mTOR, Bax, Bcl-2, AKT, TLR4, IκBα, NF-κB, caspase-3, PARP, p53	reduce the levels of OS and increase cTnT, LVEF and LVFS shortening fractions, isochoric relaxation duration, and histological and electrophysiological alteration, lessen extracellular matrix buildup and mitigate cardiac fibrosis, inhibite apoptosis and autophagy, inhibit inflammation, decreases blood levels of CK-MB, counteract DNA damage by	-	[126–130]
Ginsenoside Rg1	ginseng	SIRT 1, ATF6, IRE1, TIF1, GRP78, Beclin1, p70s6k, JNK 1	inhibit the autophagosomes, reduce ER stress	ideal tumor targeting capabilities, mitigating cardiomyocyte toxicity without sacrificing the capacity to eradicate cancer cells	[131–133]
Palmatine	Coptis chinensis	SIRT1	inhibit inflammation, OS, cardiomyocyte apoptosis	-	[134]
Tanshinone I	Salvia miltiorrhiza	Nrf2	reduce mitochondrial damage, induce OS, lower the rate of apoptosis	-	[135]
Emodin	Rhei Radix et Rhizoma	IL-1β, GSDMD	increase LVEF and LVFS, decrease serum levels of CK-MB, LDH, alleviate cardiomyocyte death and morphological disturbances, reduces mitochondrial damage, inhibit pyroptosis	-	[136]
Vanillin	Vanillin beans	caspase-3, PARP1, ERK	reduce apoptosis, inhibit OS	not counteract the efficacy of DOX in cancer	[137]
Crocin	Saffron	MDA, ROS, GSH, TAS, CAT, SOD	mitigate OS, lower levels of CK-MB and cTnI	-	[138, 139]

### Cell Therapy

#### Mesenchymal Stem Cells

Heart tissue regeneration has been the subject of much research on mesenchymal stem cell (MSC)-based tissue regeneration treatments. MSCs are more receptive to cell-based cardiac tissue regeneration therapy because they can differentiate into a range of cell lineages, including cardiomyocytes, skeletal muscle cells, osteoblasts, chondrocytes, and adipocytes. This is because mesenchymal stem cells secrete VEGF, which is secreted by mesenchymal stem cells via paracrine hepatocyte growth factor to stabilize endothelial cell barrier function [140]. DOX causes MSCs to senescence, which lowers their viability, proliferation, and paracrine actions and is linked to the development of AIC [141]. Zaki et al.’s [142] studies conducted in vitro verified that MSCs markedly decreased MDA and TNF-α while increasing VEGF, IL-10, and the Bcl2/Bax ratio. MSCs can also act on the miR-34a-SIRT1 axis, inhibit the expression of miR-34a, upregulate SIRT1, and produce the anti-aging effect of H9c2 cells. In addition, MSCs can also inhibit the expression of cell cycle-related proteins p53 and p16, increase telomere length, and telomerase activity [143]. MSCs activate the Jagged-1/Notch-1 signalling pathway by upregulating VEGF expression, resulting in inhibition of TGF-β1 release, further inhibiting DOX-induced senescence in H9c2 cells [144]. Another phase II clinical trial of BMSCs infusion (2 million cells/kg at 20, 4 and 16 months after AIC, 2 million cells/kg, 4 million cells/kg) in 3 patients with AIC at different time points (LVEF < 40%), showed a significant improvement in LVEF at 4 and 16 months after AIC [145]. Cell therapy with MSC/bone marrow mononuclear cells is effective in attenuating AIC and ameliorating cardiovascular events caused by ANT use [146].

#### Cardiac Progenitor Cells

Crucial regulators of cardiomyocyte homeostasis [147], CPCs were found to have upregulated expression of p16 inhibitor of cyclin-dependent kinase 4a [148], a well-known aging marker, in the heart tissue of the majority of DIC patients who died [149]. Additionally, the study revealed that CPCs' functional properties, including migration and differentiation, were adversely impacted. To sum up, DOX exposure significantly reduces the number of CPCs and permanently impairs their function. Premature aging of CPCs and their progeny causes the heart to have a lower capacity for regeneration, and may represent the cellular basis of DOX-induced human cardiomyopathy. Injecting enhanced green fluorescent protein-labeled CPCs into failing myocardium encourages the regeneration of cardiomyocytes and vascular structures, which improves ventricular function and animal survival [46]. Furthermore, intravenous injection of cardiac progenitor cell-derived exosomes prevents increased ROS, myocardial fibrosis, CD68 + inflammatory cell infiltration, nitric oxide synthase expression, left ventricular dysfunction, and inhibits miR-146a-5p target genes to prevent AIC. CPC exosomes also contain a variety of proteins involved in redox processes [150]. But as of right now, AIC therapy based on CPC and CPC is still in the experimental stage.

## The Current Dilemma and Challenges of AIC Prevention and Treatment

However, as far as the current research is concerned, the treatment strategies for AIC are relatively limited, the current guidelines do not strongly recommend any therapy, and the approved treatments often show conflicting results and collateral effects, which are reflected in many aspects [151].

Due to the awareness of the adverse cardiac events caused by ANTs, the use of ANTs has been restricted in clinical practice to avoid ANT-containing regimens. For example, in a randomized controlled trial of 5924 patients, no significant difference in DFS and OS was observed between docetaxel/ cyclophosphamide (TC) and doxorubicin/ cyclophosphamide/ docetaxel chemotherapy (AC-T) [152], and the latest NCCN breast cancer guidelines also eliminated epirubicin/ cyclophosphamide/ docetaxel (EC-T) regimens in favor of TC regimens. However, for patients with lobular pN2/pN3 tumors, ANT-containing chemotherapy regimens remain the preferred option.

Changing the way drugs are delivered is a fundamental way to avoid AIC, and at the same time clinical trials have also shown that the delivery method of liposome-encapsulated ANTs can avoid AIC to the greatest extent possible without compromising anti-tumor efficacy, which is currently the most mature solution. But the price is steep. Furthermore, liposomal preparations have been linked to a higher chance of a few adverse consequences, such as hand-foot syndrome and mucositis [153].

Although antioxidants are used in the prevention and treatment of AIC, there is increasing evidence that antioxidants may attenuate the anti-cancer activity of chemotherapeutic drugs [154], so it is recommended that the combination of GSH is contraindicated during chemotherapy unless ANTs causes severe hepatotoxicity and cardiotoxicity.

Statins, ACEIs/ARBs, BBs, and glucose-lowering drugs are recommended by guidelines for secondary prevention of AIC [61], but their clinical efficacy is still limited in current clinical trials, such as a previous study that evaluated 2625 patients with tumors who were scheduled to receive ANTs, and showed that close monitoring of LVEF after chemotherapy can detect almost all (98%) cases of cardiotoxicity during the first 12 months of follow-up. In addition, early treatment with ACEIs (enalapril) and receptor blockers (carvedilol or bisoprolol) normalizes cardiac function in most cases (82%), but only 11% of patients with LVEF renormalization recover completely [155]. As far as the current study is concerned, the secondary prevention of AIC is still based on studies with small samples, retrospective designs, short follow-up, or case reports.

Although DEX is considered to be the only drug for the prevention and treatment of AIC, the clinical use of DEX is limited due to the risk of secondary malignancies induced by clinical therapeutic doses [156]. And DEX may increase the risk of acute myeloid leukemia and myelodysplastic syndrome [157]. A retrospective cohort study showed that the addition of DEX to DOX resulted in a higher incidence of myelosuppression in all blood components in adjuvant patients [158]. Therefore, current clinical guidelines recommend it only as a cardioprotective agent in patients receiving high-doses of DOX [61].

At present, there is still no ideal prevention or treatment plan for AIC, but with the development of precision medicine, new therapies such as ncRNAs mesenchymal stem cell therapy and cardiac progenitor cells in the treatment of AIC are also being explored, but based on the current research, the application of ncRNAs in the field of AIC is more focused on the diagnosis of AIC, and the application in treatment is relatively limited. At the same time, the research on ncRNAs in oncology and cardiology is still at the mechanistic level, and clinical research is still limited, and the same dilemma can be seen in cell therapy. In recent years, the successful example of artemisinin in the treatment of malaria has attracted more attention to this abundant phytochemical resource [159]. And the application of phytochemicals in oncology and cardiology is also being explored. Due to their complex composition and numerous targets, some monomeric components have been used in the treatment of AIC and are a very promising therapeutic measure.

## Summary and Future Prespective

As the pathogenesis of AIC is gradually revealed, precision intervention methods based on this understanding are also updated. However, it is crucial to emphasize that caution should be exercised to ensure that cardiotoxicity is not diminished at the expense of efficacy in the development of future therapeutics and therapies before the optimal alternative drugs and regimens are utilised in clinical practice. At the same time, it is imperative to accelerate the development of alternative chemotherapy drugs for ANTs with the objective of fundamentally eliminating AIC.

## Key References

- McGowan JV, Chung R, Maulik A, et al. Anthracycline Chemotherapy and Cardiotoxicity. Cardiovasc Drugs Ther. 2017;31(1):63-75.
  - This reference is of importance because it It describes the incidence of cardiovascular events related to several anthracyclines.
- Tadokoro T, Ikeda M, Ide T, et al. Mitochondria-dependent ferroptosis plays a pivotal role in doxorubicin cardiotoxicity. JCI Insight. 2020;5(9).
  - This reference is of importance because it described that cardiotoxicity caused by doxorubicin through iron death.
- Myers CE, McGuire WP, Liss RH, et al. Adriamycin: the role of lipid peroxidation in cardiac toxicity and tumor response. Science. 1977;197(4299):165-7.
  - This reference is of importance because it described that cardiotoxicity caused by doxorubicin through iron death.
- Anversa P, Kajstura J, Leri A, et al. Life and death of cardiac stem cells: a paradigm shift in cardiac biology. Circulation. 2006;113(11):1451-63.
  - This reference is of outstanding importance because it describes the characteristics of cardiac progenitor cells.
- Pereira JD, Tosatti JAG, Simoes R, et al. microRNAs associated to anthracycline-induced cardiotoxicity in women with breast cancer: A systematic review and pathway analysis. Biomed Pharmacother. 2020;131:110709.
  - This reference is of importance because it introduces miRNAs related to AIC in heart tissue.
- Lyon AR, Lopez-Fernandez T, Couch LS, et al. 2022 ESC Guidelines on cardio-oncology developed in collaboration with the European Hematology Association (EHA), the European Society for Therapeutic Radiology and Oncology (ESTRO) and the International Cardio-Oncology Society (IC-OS). Eur Heart J. 2022;43(41):4229-361.
  - This reference is of outstanding importance because it introduces the high-risk doses of different kinds of anthracyclines.
- Pedicino D, Patrono C. Adverse effects of anthracyclines: does atorvastatin STOP-CArdiotoxicity? Eur Heart J. 2023;44(43):4506-7.
  - This reference is of importance because it shows that atorvastatin can effectively reduce the incidence of AIC.
- Zhong Z, Gao Y, Zhou J, et al. Inhibiting mir-34a-5p regulates doxorubicin-induced autophagy disorder and alleviates myocardial pyroptosis by targeting Sirt3-AMPK pathway. Biomed Pharmacother. 2023;168:115654.
  - This reference is of outstanding importance because it shows the miR-34-5p regulates autophagy and ROS production by down-regulating SIRT3 pathway.

## Data Availability

No datasets were generated or analysed during the current study.

## Abbreviations

CTR-CVT: Cancer therapy-related cardiovascular toxicity
ANT: Anthracycline
AIC: ANT-induced cardiotoxicity
DOX: Doxorubicin
NCCN: The national comprehensive cancer network
OS: Oxidative stress
Top2: Topoisomerase II
ROS: Reactive oxygen species
RNS: Reactive nitrogen species
NOS: Nitric oxide synthase
NADPH: Nicotinamide adenine dinucleotide phosphate
NOX: NADPH oxidase
NADH: Nicotinamide-adenine dinucleotide
ND: NADH dehydrogenase
ATP: Adenosinetriphosphate
DIC: DOX-induced cardiotoxicity
WT: Wild-typ
nNOS: Neuronal NOS
eNOS: Endothelial NOS
iNOS: Injury-inducible NOS
4-HNE: 4-Hydroxynonenal
GPX4: Glutathione peroxidase 4
ER: Endoplasmic reticulum
CASPASE: Cysteinyl aspartate specific proteinase
IL: Interleukin
TLR: Toll-like receptor
TNF: Tumor necrosis factor
CPC: Cardiac progenitor cell
ncRNA: Non-coding RNA
miRNA: MicroRNA
lncRNA: Long non-coding RNA
circRNA: Circle RNA
RAAS: The renin–angiotensin–aldosterone system
AngII: Angiotensin II
AT1: AngII-1A receptor
DEX: Dexrazoxane
ESC: European Society of Cardiology
DDS: Drug delivery system
NP: Nanoparticle
PLGA-NP: Poly(lactic-co-glycolic acid)-grafted silica NP
MDA: Malondialdehyde
SOD: Superoxide dismutase
Bcl-2: B-cell lymphoma-2
Bax: Bcl-2 assaciated X protein
NQO1: NAD(P)H dehydrogenase quinone 1
GSH: Glutathione
CAT: Catalase
LVEF: Left ventricular ejection fraction
BNP: Brain natriuretic peptide
CK: Creatine kinase
CK-MB: Creatine kinase-MB isoenzyme
LDH: Lactate dehydrogenase;
ADR-925, N: N'-[(1S)-1-methyl-1,2-ethanediyl]-bis [(N-(2-amino-2-oxoethyl)]glycine
MAPK: Mitogen-activated protein kinase
NF-κB: Nuclear factor-k-gene binding
STAT: Signal transducer and activator of transcription
p-Akt: Phosphorylated protein kinase B
p-JNK: Phosphorylated c-Jun amino-terminal kinase
p-ERK: Phosphorylated extracellular signal-regulated kinase
BB: Beta-blockers
ARB: Angiotensin receptor blocker
ACEI: Angiotensin-converting enzyme inhibitor
PI3K: Phosphatidylinositol 3-kinase
ATF: Activating transcription factor
elF: Eukaryotic initiation factor
CHOP: C/EBP homologous protein
PERK: Pancreatic endoplasmic reticulum kinase
GRP78: Glucose-regulated protein 78
AMPK: Adenosine monophosphat-activated protein kinase
mTOR: Mammalian target of rapamycin
TGF: Transforming growth factor
SGLT: Sodium-glucose cotransporter
DAPA: Dapagliflozin
Nrf: Nuclear respiratory factor
SMAD: Small mothers against decapentaplegic
SIRT: Sirtuins
PPAR: Peroxisome proliferator-activated receptors
XPO 1: Exportin-1
HAX-1: Hematopoietic cell-specific protein-associated protein X-1
NEAT: Nuclear paraspeckle assembly transcript
ATG4a: Autophagy related 4A cysteine peptidase gene
MALAT: Metastasis-associated lung adenocarcinoma transcript
PAE: Paeonol
Mfn: Mitofusin
PKC: Protein kinase C
SCU: Scutellarin
cTnT: Cardiac troponin T
LVFS: Left ventricular fractional shortening
AST: Aspartate aminotransferase
ALT: Alanine aminotransferase
PLT: Palmitine
Tan: Tanshinone
EMO: Emodin
MSC: Mesenchymal stem cell
